# Targeted ablation of the cellular inhibitor of apoptosis 1 (cIAP1) attenuates denervation-induced skeletal muscle atrophy

**DOI:** 10.1186/s13395-019-0201-6

**Published:** 2019-05-24

**Authors:** Neena Lala-Tabbert, Rim Lejmi-Mrad, Kristen Timusk, Marina Fukano, Janelle Holbrook, Martine St-Jean, Eric C. LaCasse, Robert G. Korneluk

**Affiliations:** 1 Apoptosis Research Centre, Children’s Hospital of Eastern Ontario Research Institute, 401 Smyth Road, Ottawa, ON K1H 8L1 Canada; 2 Department of Biochemistry, Microbiology and Immunology, Faculty of Medicine, University of Ottawa, Ottawa, ON K1H 8M5 Canada; 3 Department of Cellular and Molecular Medicine, Faculty of Medicine, University of Ottawa, Ottawa, ON K1H 8M5 Canada

**Keywords:** cIAP1, NF-κB, Muscle atrophy, MuRF1, Atrogin-1, Denervation

## Abstract

**Background:**

Skeletal muscle atrophy is a pathological condition that contributes to morbidity in a variety of conditions including denervation, cachexia, and aging. Muscle atrophy is characterized as decreased muscle fiber cross-sectional area and protein content due, in part, to the proteolytic activities of two muscle-specific E3 ubiquitin ligases: muscle RING-finger 1 (MuRF1) and muscle atrophy F-box (MAFbx or Atrogin-1). The nuclear factor-kappa B (NF-κB) pathway has emerged as a critical signaling network in skeletal muscle atrophy and has become a prime therapeutic target for the treatment of muscle diseases. Unfortunately, none of the NF-κB targeting drugs are currently being used to treat these diseases, likely because of our limited knowledge and specificity, for muscle biology and disease. The cellular inhibitor of apoptosis 1 (cIAP1) protein is a positive regulator of tumor necrosis factor alpha (TNFα)-mediated classical NF-κB signaling, and cIAP1 loss has been shown to enhance muscle regeneration during acute and chronic injury.

**Methods:**

Sciatic nerve transection in wild-type, cIAP1-null and Smac mimetic compound (SMC)-treated mice was performed to investigate the role of cIAP1 in denervation-induced atrophy. Genetic in vitro models of C2C12 myoblasts and primary myoblasts were also used to examine the role of classical NF-κB activity in cIAP1-induced myotube atrophy.

**Results:**

We found that cIAP1 expression was upregulated in denervated muscles compared to non-denervated controls 14 days after denervation. Genetic and pharmacological loss of cIAP1 attenuated denervation-induced muscle atrophy and overexpression of cIAP1 in myotubes was sufficient to induce atrophy. The induction of myotube atrophy by cIAP1 was attenuated when the classical NF-κB signaling pathway was inhibited.

**Conclusions:**

These results demonstrate the cIAP1 is an important mediator of NF-κB/MuRF1 signaling in skeletal muscle atrophy and is a promising therapeutic target for muscle wasting diseases.

**Electronic supplementary material:**

The online version of this article (10.1186/s13395-019-0201-6) contains supplementary material, which is available to authorized users.

## Background

In a variety of diseases, such as HIV/AIDS and cancer, skeletal muscle atrophy is a significant cause of human morbidity and death. Muscle atrophy is the outcome of adaptive and pathological circumstances, in which anabolic processes that promote synthesis are counterbalanced by catabolic processes that promote proteolysis. Such circumstances can be induced by various intrinsic and exogenous factors, including denervation, aging (sarcopenia), systemic inflammation, muscle disuse (as in cast immobilization and prolonged bed rest), and cachexia [1]. The specific biochemical mechanisms leading to atrophy may involve inhibition of protein synthesis, acceleration of proteasomal degradation, or a combination of both [1, 2]. The identification of inducible muscle-specific ubiquitin ligases that are key to these proteolytic processes, namely muscle RING finger-1 (MuRF1) and muscle atrophy F-Box 1 (MAFbx1, or Atrogin-1) [3, 4], allowed the study of atrophy as an outcome of signal transduction events that could be pharmacologically manipulated for therapeutic purposes. These ubiquitin ligases are regulated by a number of cellular signaling pathways including those involving nuclear factor kappa-light-chain-enhancer of activated B cells (NF-κB) transcription factors [5, 6].

The NF-κB pathway is critically important to cytokine and inflammatory signaling networks in skeletal muscle biology and disease [7]. Excessive activation of the classical pathway is a hallmark and aggravating feature of various musculoskeletal disorders including cachexia and denervation [5, 6, 8, 9]. For example, genetic or pharmacological inhibition of IKKβ/NF-κB reduced MuRF1 expression and attenuated denervation- and tumor-induced muscle atrophy in mice [5, 6]. Furthermore, direct activation of the IKK complex through transient overexpression of a constitutively active form of IKKβ or IKKα consistently led to skeletal muscle atrophy [10]. Due to its importance in muscle pathology, the NF-κB signaling pathway is a therapeutic target for a variety of diseases and, consequently, numerous inhibitors have been evaluated in human trials [11, 12]. Unfortunately, none of these drugs are currently being used to treat skeletal muscle atrophy, likely because our ability to specifically target NF-κB in muscle is hampered by our limited understanding of its complex regulation.

The inhibitors of apoptosis (IAP) proteins are a family of animal apoptosis regulators that were originally identified while searching for candidate genes responsible for spinal muscular atrophy [13, 14]. The IAPs are characterized by the presence of one or more baculovirus IAP repeat (BIR) domains which participate in caspase inhibition and signal transduction [15–18]. The best-studied IAPs include cellular IAP1 (cIAP1) and cIAP2, both which contain a C-terminal really interesting new gene (RING) zinc finger that acts as an E3 ubiquitin ligase in addition to their three BIR domains [15, 19]. Interestingly, cIAP1 and cIAP2 have emerged as key regulators of both classical and alternative arms of the NF-κB pathway [18, 20–22].

Given the central roles of the cIAPs in regulating NF-κB signaling, we investigated the role of cIAP1 in skeletal muscle regeneration [23, 24]. We focused on cIAP1, since its redundant paralog cIAP2 is not normally expressed in muscle cells [20]. We observed that loss of cIAP1 dramatically increased myoblast fusion, both in vitro to form myotubes and in vivo to regenerate muscle fibers [24]. In mdx mice, which exhibit chronic muscle degeneration due to the absence of the structural protein dystrophin, the loss of cIAP1 increased the resistance of muscle to damage and improved muscle function [23]. Our mechanistic analyses revealed that the alternative NF-κB pathway is a positive regulator of myoblast fusion and that cIAP1 downregulation increases myoblast fusion through the de-repression of this pathway [22–24].

In the present study, we aimed to elucidate the function of cIAP1 in skeletal muscle atrophy. We found that cIAP1 expression is upregulated during sciatic nerve denervation-induced muscle atrophy. Using a complete cIAP1 knockout mouse, we found that genetic ablation of cIAP1 can attenuate denervation-induced atrophy in most muscles. Furthermore, overexpression of cIAP1 in myotubes was sufficient to induce myotube atrophy through the activation of the inhibitor of NF-κB kinase subunit beta (IKKβ)/NF-κB/MuRF1 pathway. Altogether, our findings establish cIAP1 as a positive regulator of skeletal muscle atrophy and suggest that the suppression of cIAP1 could serve as a potential therapy for skeletal muscle atrophy.

## Methods

### Animal care

Mice were bred and handled as recommended by the guidelines established by the University of Ottawa Animal Care Veterinary Service and the Canadian Council on Animal Care. C57BL/6 mice aged 6–8 weeks were obtained from Jackson Laboratories. The cIAP1-null mice and cIAP2-null mice were generated by the Ashwell lab and Korneluk lab, respectively, and genotyped accordingly [25, 26]. All animals were housed in a controlled facility (22 °C with 30% relative humidity on a 12-h light/dark cycle) and provided with food and water ad libitum.

Denervation was performed in the right hind limb, and a sham operation was performed in the left hind limb. The sciatic nerve was exposed through a skin incision, and a 5–10-mm-long segment of the sciatic nerve was removed. The skin incision was closed using 4-0 nylon sutures. For the sham operation, identical surgical procedures were performed, except for the nerve transection. In order to investigate if Smac mimetic compounds (SMCs) reduce muscle atrophy, mice were treated with vehicle (30% 0.1N HCl + 70% 100 mM NaOAc) or LCL161 (75 mg/kg, Novartis) by oral gavage at 4, 7, and 11 days post-denervation. Mice were sacrificed 7 or 14 days post-denervation and the left and right tibialis anterior (TA), gastrocnemius (Gas), soleus (Sol), and extensor digitorum longus (EDL) were collected, weighed, embedded in Tissue-Tek OCT compound, and flash frozen in isopentane cooled by liquid nitrogen. Cryosections (8-μm thick) were stained with hematoxylin and eosin (H&E) (Sigma).

### C2C12 and primary myoblast culture

C2C12 myoblasts (ATCC) were cultured and maintained in DMEM (Fisher Scientific) supplemented with 10% heat-inactivated fetal bovine serum (FBS; Sigma) (growth media, GM). To induce differentiation, C2C12 myoblasts were switched to DMEM containing 2% heat-inactivated horse serum (HS; Sigma) for 4 days.

Primary myoblasts were isolated as described previously [27]. Briefly, hind limb muscles of C57BL/6 mice (6 to 8 weeks of age) mice were dissected and digested in a 0.2% Collagenase Type 2 solution (Worthington Biochemical). After digestion, the muscle slurry was filtered through a 70-uM cell strainer to remove undigested muscle. Cells were washed with serum-free media (SFM) and then enriched for myoblasts by magnetic-activated cell sorting (MACS). Primary myoblasts were grown on Matrigel-coated plates in DMEM containing 20% heat-inactivated FBS, 10% heat-inactivated HS, and supplemented with 10 ng/ml basic fibroblast growth factor (bFGF) and 2 ng/ml human growth factor (HGF) (PeproTech Inc.). Differentiation was induced by changing the media of confluent myoblasts to DMEM containing 2% heat-inactivated FBS and 10% heat-inactivated HS for 48 h.

### Adenovirus infection and rescue experiments

For adenovirus infections, C2C12 and primary myoblasts were induced to differentiate for 4 or 2 days, respectively. Adenovirus expressing green fluorescent protein (GFP) or human cIAP1 was added to mature myotubes at a multiplicity of infection (MOI) of 400. Myotubes were collected 24 h after infection.

For siRNA rescue experiments, nascent C2C12 myotubes (DM day 2) were transfected with non-targeting siRNA or siRNA targeting murine IKKβ (Dharmacon) using Lipofectamine RNAiMAX reagent (Invitrogen) following the manufacturer’s instructions. Two days after transfection, myotubes were infecting with GFP or human cIAP1 adenovirus as described above.

For pharmacological rescue experiments, fully differentiated primary myotubes (DM day 2) were treated with TNFα (10 ng/ml, R&D Systems) or dexamethasone (50 uM, Sigma Aldrich) in the absence or presence of the SMC, LCL161 (500 nM, Novartis). Myotubes were collected 24 h after treatment.

### Western analysis

Protein extracts were harvested from myotubes or gastrocnemius muscle with protease and phosphatase inhibitors, resolved on an 8 or 10% SDS-PAGE gel, and transferred to a nitrocellulose membrane. Total protein staining was performed using REVERT™ total protein stain (LI-COR) following the manufacturer’s protocol to normalize the protein levels. Membranes were then probed with specific antibodies: Atrogin-1 (Abcam, ab168372), MuRF1 (ECM Biosciences, MP3401), myosin heavy chain (DSHB, MF-20), anti-rat IAP1 which detects mouse, rat and human cIAP1 and cIAP2 proteins as well (cIAP1/2; MBL International Corporation, Cy-P1041), phospho-p65 (Cell Signaling Technology, #3033), total p65 (Cell Signaling Technology, #3034), IKKβ (Cell Signaling Technology, #2370), and GAPDH (Advanced ImmunoChemical Inc., 2-RGM2). Alexa Fluor 680 (Invitrogen) or IRDye® 800 (LI-COR) were used to detect the primary antibodies and fluorescent signals were detected using the Odyssey Infrared Imaging System (LI-COR).

### Immunofluorescence

C2C12 and primary myotubes were fixed in ice-cold 100% methanol, washed three times in phosphate-buffered saline (PBS), and permeabilized with PBS containing 0.5% Triton X-100 prior to incubation with primary antibody overnight at 4 °C. The primary antibodies used were myosin heavy chain (DSHB, MF-20) and GFP (Abcam, ab290). Secondary antibodies conjugated to a fluorescent dye (Alexa Flour 488 or 546; Invitrogen) were used, and nuclei were counterstained with DAPI (0.5 μg/ml).

### Image acquisition

Digital images of fluorescently stained myotubes and H&E stained muscle sections were acquired using an inverted Olympus IX51 microscope, an Olympus microscope DP72 digital camera, and Olympus cellSens Entry imaging software. Images were taken at room temperature and were composed and edited in Paint.net.

### Statistical analysis

Statistical analysis was performed using the GraphPad Prism Software. A two-tailed student’s *t* test was performed when comparing a single experimental condition to the control condition. Significance is indicated as *< 0.05, **< 0.01, or ***< 0.001. A single factor ANOVA was used to determine significance between three or more experimental conditions, and a Bonferroni’s post hoc test was used to compare conditions when significance was determined by the one-way ANOVA. Means with no common letters are significantly different (*p* < 0.05) from one another. All experiments are representative of a minimum of three biological replicates and are presented as the mean ± standard error mean (SEM).

## Results

### Expression of cIAP1 is upregulated in atrophying muscle

**Fig. 1:**
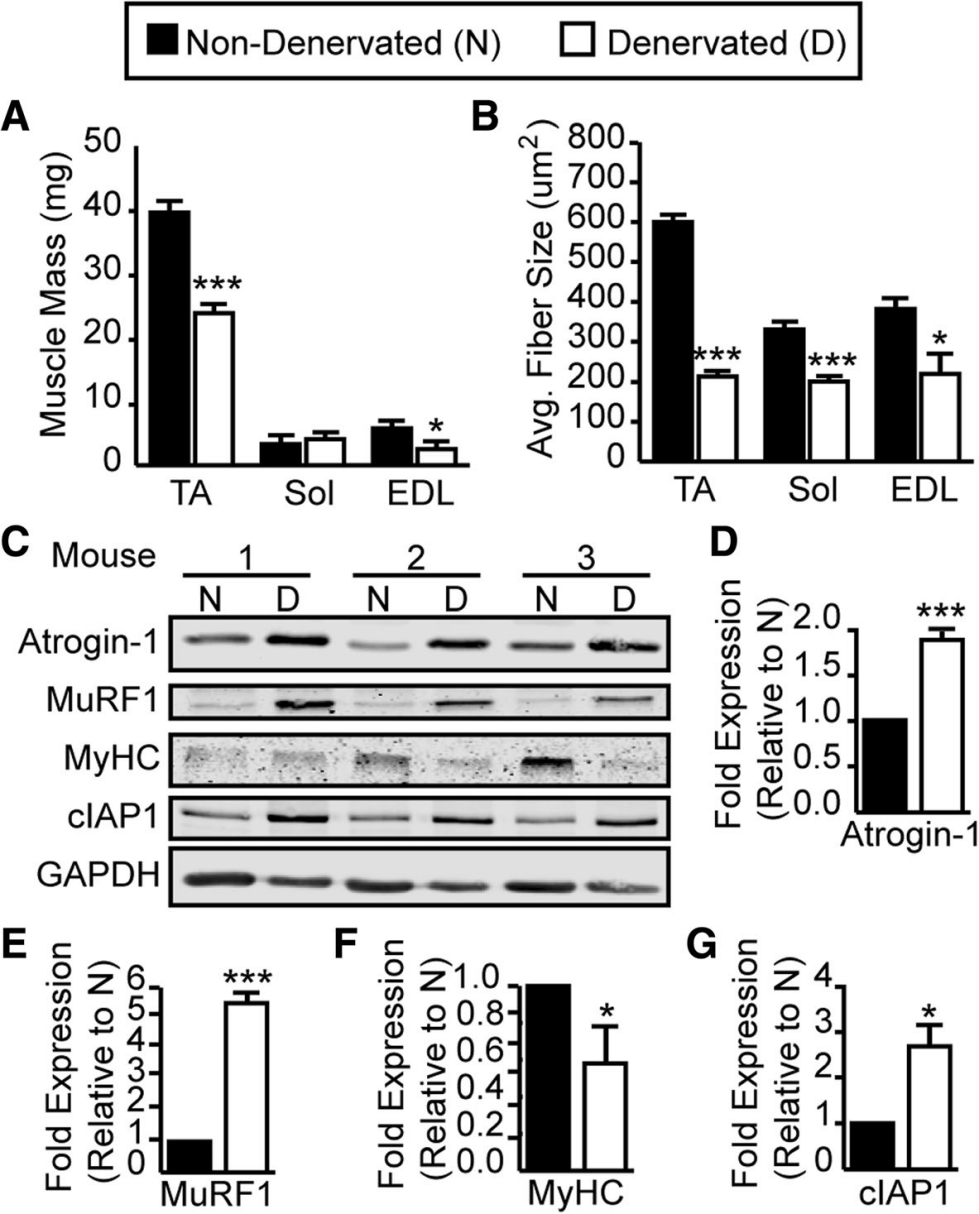
cIAP1 expression is induced in denervation-induced muscle atrophy. A small (5–10 mm) piece of the right sciatic nerve was removed to induce denervation of the entire right hind limb of 6-week-old female C57BL/6 mice. The contralateral leg served as an internal control. Mice were sacrificed 14 days post denervation. **a** Muscle mass of tibialis anterior (TA), soleus (Sol) and extensor digitorum longus (EDL) from non-denervated (black bars) or denervated (white bars) hind limbs. **b** Average fiber size of muscle fibers from non-denervated or denervated TA, Sol, and EDL muscles. **c** Representative Western blots of Atrogin-1, MuRF1, MyHC, cIAP1, and GAPDH protein expression in Gas muscle from non-denervated (N) and denervated (D) hind limbs. Quantification of **d** Atrogin-1, **e** MuRF1, **f** MyHC, and **g** cIAP1 protein expression normalized with total protein and relative to non-denervated (N) muscle. Data is the mean ± SEM, *n* = 4, *p < 0.05, ***p < 0.001

To investigate the potential role of cIAP1 in skeletal muscle atrophy, we used a robust denervation model in which the sciatic nerve was transected to induce muscle atrophy of the hind limbs. The contralateral leg served as a non-denervated control. We confirmed that 14 days of denervation in wild-type mice reduced muscle mass and cross-sectional area of the tibialis anterior (TA), soleus (Sol), and extensor digitorum longus (EDL) (Fig. 1a, b). Western blot analysis of protein extracts isolated from non-denervated (N) and denervated (D) gastrocnemius muscle demonstrated significant upregulation of Atrogin-1 and MuRF1, atrogenes that are critically involved in muscle atrophy. As well, significant downregulation of myosin heavy chain (Fig. 1c–f) was found. Thus, transection of the sciatic nerve induced skeletal muscle atrophy.

Using the denervation-induced atrophy model, we next examined whether there was a change in cIAP1 protein levels in denervated muscle compared to non-denervated muscle. Interestingly, western blot analysis demonstrated a significant upregulation of cIAP1 protein expression (2.7-fold) in denervated muscle compared to non-denervated controls (Fig. 1c, g). We used an anti-RIAP1 antibody that detects both cIAP1 and cIAP2; however, we did not see the expression of cIAP2, a complementary paralog of cIAP1, in control or atrophying muscle, consistent with what we have observed previously [20]. Taken together, these results suggest that cIAP1 expression is substantially upregulated during denervation-induced atrophy.

### cIAP1 controls myotube atrophy in vitro

**Fig. 2:**
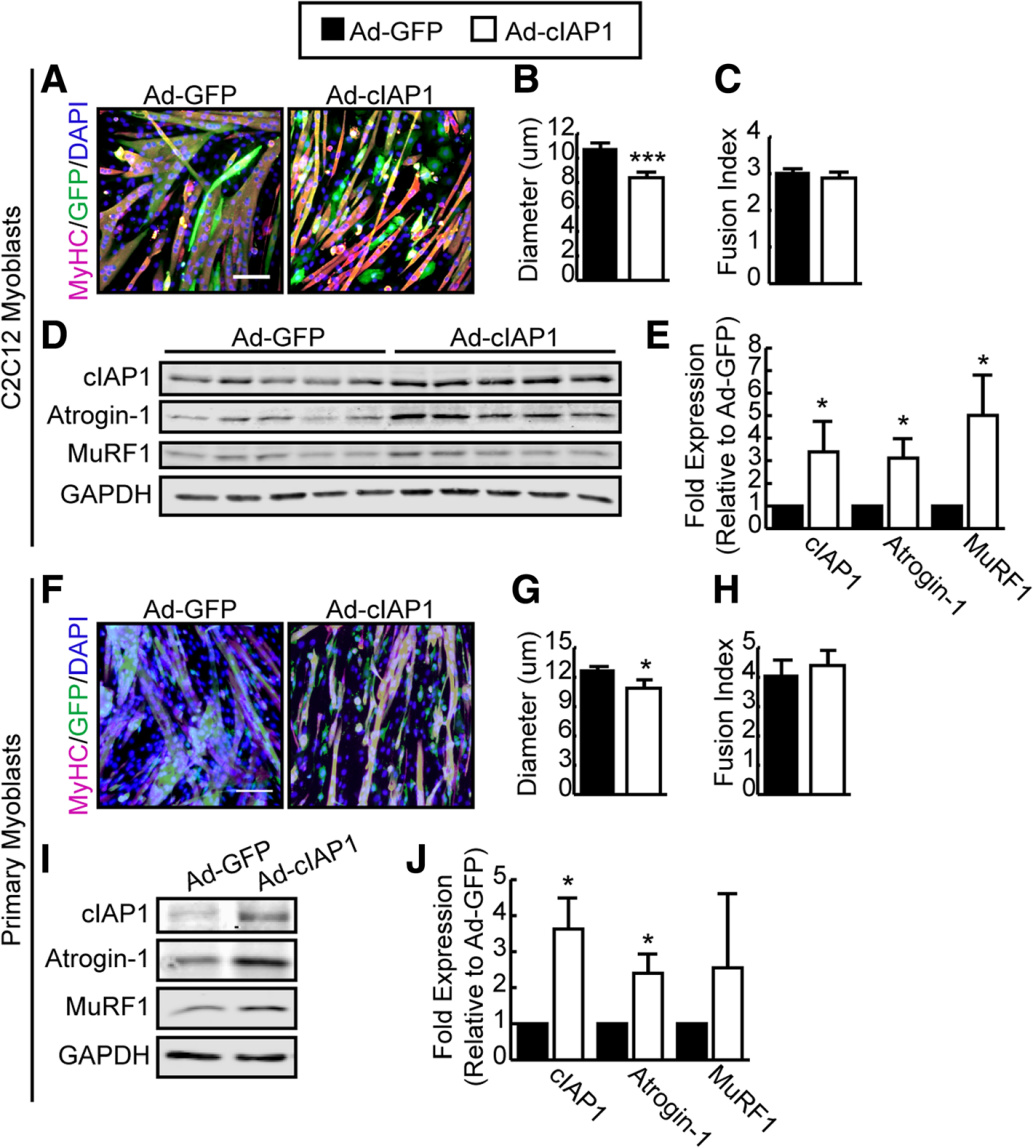
cIAP1 is sufficient to induce muscle atrophy in vitro. **a** C2C12 myoblasts were allowed to differentiate for 4 days to produce myotubes. Myotubes were infected with adenovirus expressing GFP (Ad-GFP) or cIAP1 (Ad-cIAP1) for 24 h at an MOI of 400. Myotubes were stained with MyHC (pink) and GFP (green), and nuclei were counterstained with DAPI (blue). Representative images are shown. Scale bar =50um. **b** Myotube diameter of MyHC^+^/GFP^+^ myotubes stained as in **a** (*n* = 5). **c** Fusion index (number of nuclei/myotubes) of MyHC^+^/GFP^+^ myotubes stained as in **a** (*n* = 5). **d** Representative western blots of cIAP1, Atrogin-1, MuRF1, and GAPDH expression in cells cultured as described in **a**. **e** Quantification of cIAP1, Atrogin-1, and MuRF-1 protein expression normalized with total protein and relative to Ad-GFP infected C2C12 myotubes (*n* = 5). **f** Primary myoblasts were isolated from C57BL/6 mice and allowed to differentiate for 48 h to produce myotubes. Myotubes were infected with adenovirus expressing GFP (Ad-GFP) or cIAP1 (Ad-cIAP1) for 24 h at an MOI of 400. Myotubes were stained with MyHC (pink) and GFP (green) and nuclei were counterstained with DAPI (blue). Representative images are shown. Scale bar = 50 um. **g** Myotube diameter of MyHC^+^/GFP^+^ myotubes stained as in **e** (*n* = 3). **h** Fusion index (number of nuclei/myotubes) of MyHC^+^/GFP^+^ myotubes stained as in **e** (*n* = 3). **i** Representative western blots of cIAP1, Atrogin-1, MuRF-1, and GAPDH expression in cells cultured as described in **e**. **j** Quantification of cIAP1, Atrogin-1, and MuRF-1 protein expression normalized with total protein and relative to Ad-GFP infected primary myotubes (*n* = 3). Data is the mean ± SEM, *p < 0.05, **p < 0.01, ***p < 0.001

To examine the role of cIAP1 in promoting muscle atrophy, fully differentiated C2C12 myotubes were infected with adenovirus expressing GFP (Ad-GFP) or cIAP1 (Ad-cIAP1) for 24 h. Cultures were then fixed and immunostained for GFP and myosin heavy chain (MyHC) to measure myotube diameter (Fig. 2a, b). C2C12 myotubes that were infected with cIAP1 had significantly smaller myotubes (8.4 μm) compared to GFP-infected myotubes (10.8 μm) (Fig. 2b) suggesting that overexpression of cIAP1 is sufficient to induce myotube atrophy in vitro. Since we have previously shown that loss of cIAP1 in myoblasts enhances myoblast fusion [24], we examined whether the decrease in myotube diameter was due to loss of cytoplasm or a decrease in the fusion of nuclei. Fusion index was assessed, and we found that there was no difference in the number of nuclei per myotube in GFP- and cIAP1-infected myotubes (Fig. 2c) suggesting that cIAP1 induces myotubes atrophy by reducing cytoplasmic volume. Western blot analysis of protein extracts from these cultures confirmed the increase in cIAP1expression (Fig. 2d, e). Overexpression of cIAP1 was also sufficient to significantly elevate Atrogin-1 (threefold) and MuRF1 (fivefold) protein expression in wild-type myotubes (Fig. 2d, e).

To confirm these findings, we isolated myoblasts from wild-type mice and induced them to differentiate and infected differentiated myotubes with adenovirus expressing GFP (Ad-GFP) or cIAP1 (Ad-cIAP1). Consistent with our C2C12 data, we saw that overexpression of cIAP1 reduced myotube diameter with no change in fusion index and significantly increased Atrogin-1 protein expression in primary cells as well (Fig. 2f–j). Although there was a trend towards increased MuRF1 expression in cIAP1-infected myotubes, this upregulation did not reach statistical significance (Fig. 2i, j). Taken together, these results suggest that overexpression of cIAP1 is sufficient to promote myotube atrophy in vitro.

### Genetic ablation of cIAP1 attenuates denervation-induced atrophy

**Fig. 3:**
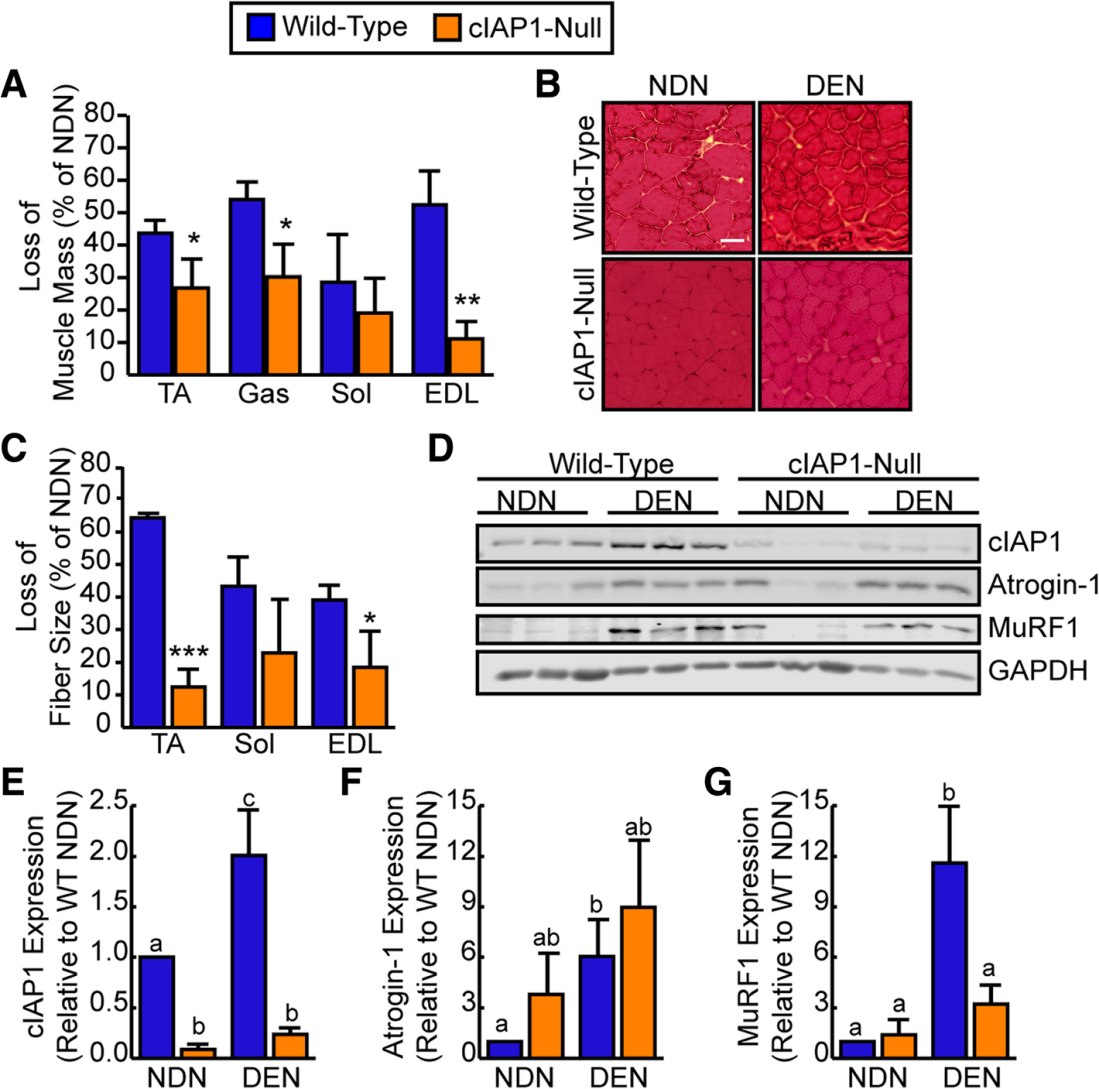
Genetic ablation of cIAP1 attenuates denervation-induced atrophy. A small (5–10 mm) piece of the right sciatic nerve was removed to induce denervation of the entire right hind limb of 6-week-old C57BL/6 mice (blue bars) or sex- and age-matched cIAP1-null mice (orange bars). The contralateral leg served as an internal control. Mice were sacrificed 14 days post-denervation. **a** Loss of muscle mass as a percentage of non-denervated control leg mass of tibialis anterior (TA), gastrocnemius (Gas), soleus (Sol), and extensor digitorum longus (EDL) (*n* = 4). **b** Representative images of TA muscle sections stained with H&E from mice described above. Scale bar = 20um. **c** Loss in TA, Sol, and EDL fiber size as a percentage of non-denervated control leg cross-sectional area (*n* = 4). **d** Representative western blots of cIAP-1, Atrogin-1, MuRF1, and GAPDH protein expression in Gas muscle from non-denervated (NDN) and denervated (DEN) hind limbs of C57BL/6 and cIAP1-null mice. Quantification of **e** cIAP1, **f** Atrogin-1, and **g** MuRF-1 protein expression normalized with total protein and relative to wild-type non-denervated (WT NDN) muscle (*n* = 3). Data is the mean ± SEM, *p < 0.05, **p < 0.01, and ***p < 0.001, means with no common letters are significantly different from each other (*p* < 0.05)

Since cIAP1 is upregulated in denervated muscle, and since cIAP1 overexpression is sufficient to induce myotube atrophy in vitro, we next examined whether cIAP1 is required for denervation-induced muscle atrophy in vivo. Accordingly, we denervated the right hind limbs of cIAP1-null mice and followed the mice for 7 or 14 days post-surgery. Sex- and age-matched wild-type mice were used as the controls. Examination of the cross-sectional area at 7 days post-denervation showed that there was no difference in fiber size loss between wild-type and cIAP1-null TA (Additional file 1: Figure S1); however, gross analysis showed that loss of muscle mass of the TA, gastrocnemius, and EDL were significantly reduced in cIAP1-null animals compared to wild-type animals (Fig. 3a). We also assessed the fiber cross-sectional area of the TA, soleus, and EDL after staining muscle sections with hematoxylin and eosin (Fig. 3b, c) and found that the decrease in mean fiber cross-sectional area after denervation was reduced in the TA (12% compared to 64%) and EDL (18% compared to 39%) of cIAP1-null mice compared to wild-type mice (Fig. 3c). Interestingly, the loss of cIAP1 did not appear to significantly attenuate denervation-induced atrophy in the soleus muscle, although a slight trend was seen (Fig. 3a, c).

We then examined whether the loss of cIAP1 could inhibit activation of the ubiquitin-proteasome system (UPS) by evaluating Atrogin-1 and MURF1 protein expression in mice. Total protein was extracted from non-denervated (NDN) and denervated (DEN) gastrocnemius of wild-type and cIAP1-null mice. Western blot analysis confirmed cIAP1 upregulation (twofold) in denervated compared to non-denervated wild-type muscle as well as the loss of cIAP1 expression in the cIAP1-null animals (Fig. 3d, e). We did not see compensation of cIAP2 for cIAP1 in the skeletal muscle of cIAP1-null mice (Fig. 3d). Interestingly, loss of cIAP1 expression significantly inhibited upregulation of MuRF1 protein levels in denervated muscle of cIAP1-null mice (Fig. 3d, g) but had no effect on the upregulation of Atrogin-1 expression (Fig. 3d, f). These results suggest that cIAP1 is necessary for denervation-induced muscle atrophy and for activation of the UPS during muscle atrophy.

**Fig. 4:**
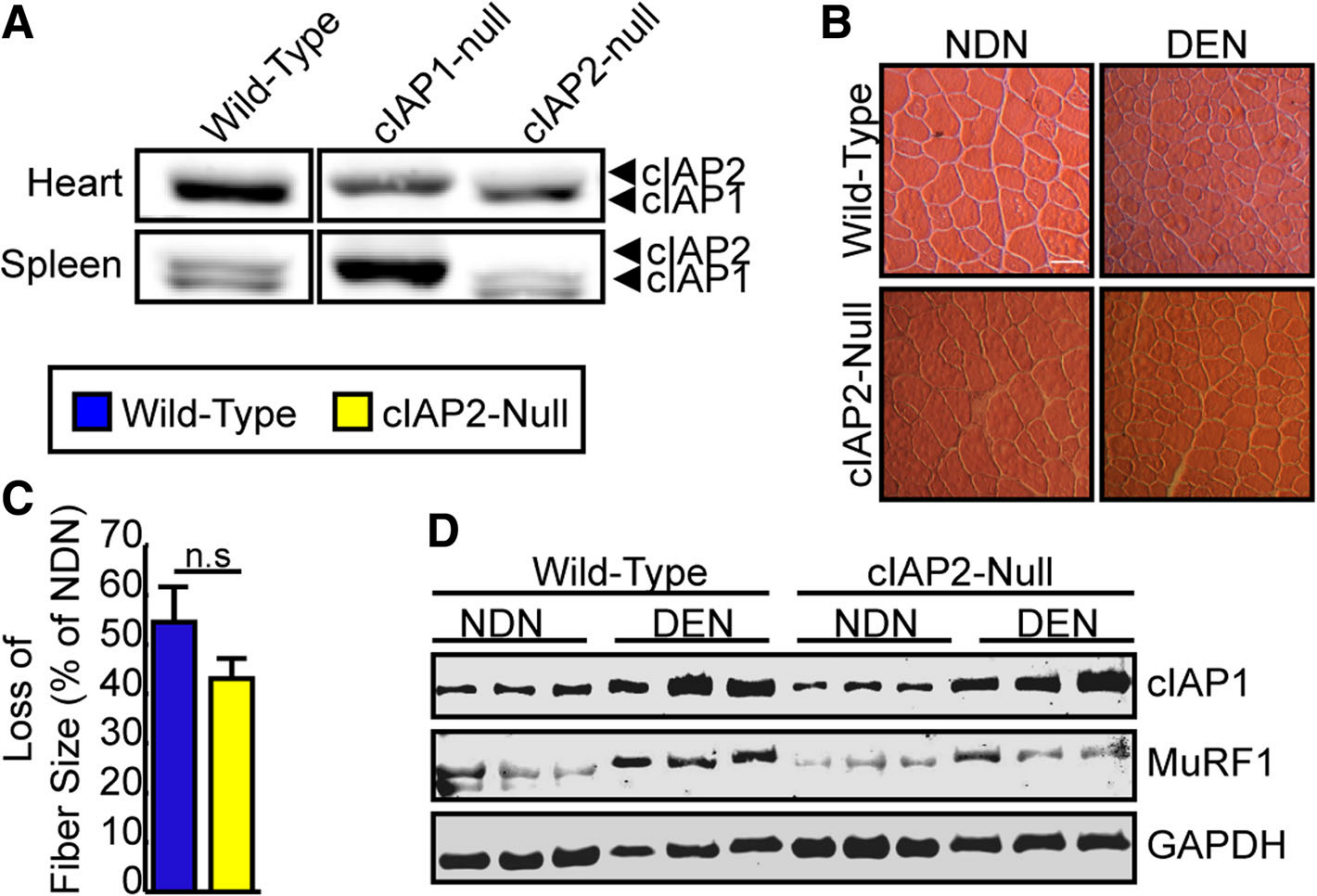
Genetic ablation of cIAP2 does not attenuate denervation-induced atrophy. A small (5–10 mm) piece of the right sciatic nerve was removed to induce denervation of the entire right hind limb of 6-week-old C57BL/6 mice (blue bars) or sex- and age-matched cIAP2-null mice (yellow bars). The contralateral leg served as an internal control. Mice were sacrificed 14 days post-denervation. **a** Representative Western blots of cIAP1 and cIAP2 protein expression of the heart and spleen of C57BL/6, cIAP1-null, and cIAP2-null mice. **b** Representative images of TA muscle sections stained with H&E from mice described above. Scale bar = 20 um. **c** Loss in TA fiber size as a percentage of non-denervated cross-sectional area (*n* = 3). **d** Representative Western blots of cIAP1, MuRF1, and GAPDH protein expression in gastrocnemius muscle from non-denervated (NDN) and denervated (DEN) hind limbs of C57BL/6 and cIAP2-null mice. Data is the mean ± SEM, n.s. not significant

Since cIAP2 expression and function have been shown to compensate for the loss of cIAP1 in other tissues and has been shown to increase in other tissues of cIAP1-null mice [20, 25, 28], we examined whether loss of cIAP2 could also attenuate denervation-induced atrophy in a muscle-independent manner. The phenotype of cIAP2-null mice was confirmed by Western blot analysis of protein extracted from the heart and spleen (Fig. 4a). Next, we denervated the right hind limbs of cIAP2-null mice and wild-type mice for 14 days. The TA muscles were isolated, frozen, sectioned, and stained with H&E to measure mean cross-sectional area (Fig. 4b, c). Loss of cIAP2 did not attenuate muscle atrophy as demonstrated by the same decrease in fiber size in both wild-type (54%) and cIAP2-null (43%) muscles (Fig. 4c). Western blot analysis of protein extracts from the gastrocnemius muscle of wild-type and cIAP2-null animals demonstrated an increase in cIAP1 and MuRF1 protein expression in denervated muscle of wild-type and null animals (Fig. 4d), suggesting that cIAP2 is not expressed in skeletal muscle and does not play a role in denervation-induced muscle atrophy through other non-muscle cell types that express cIAP2, such as macrophages expressing cell types.

### cIAP1 mediates muscle atrophy through activation of classical NF-κB signaling

**Fig. 5:**
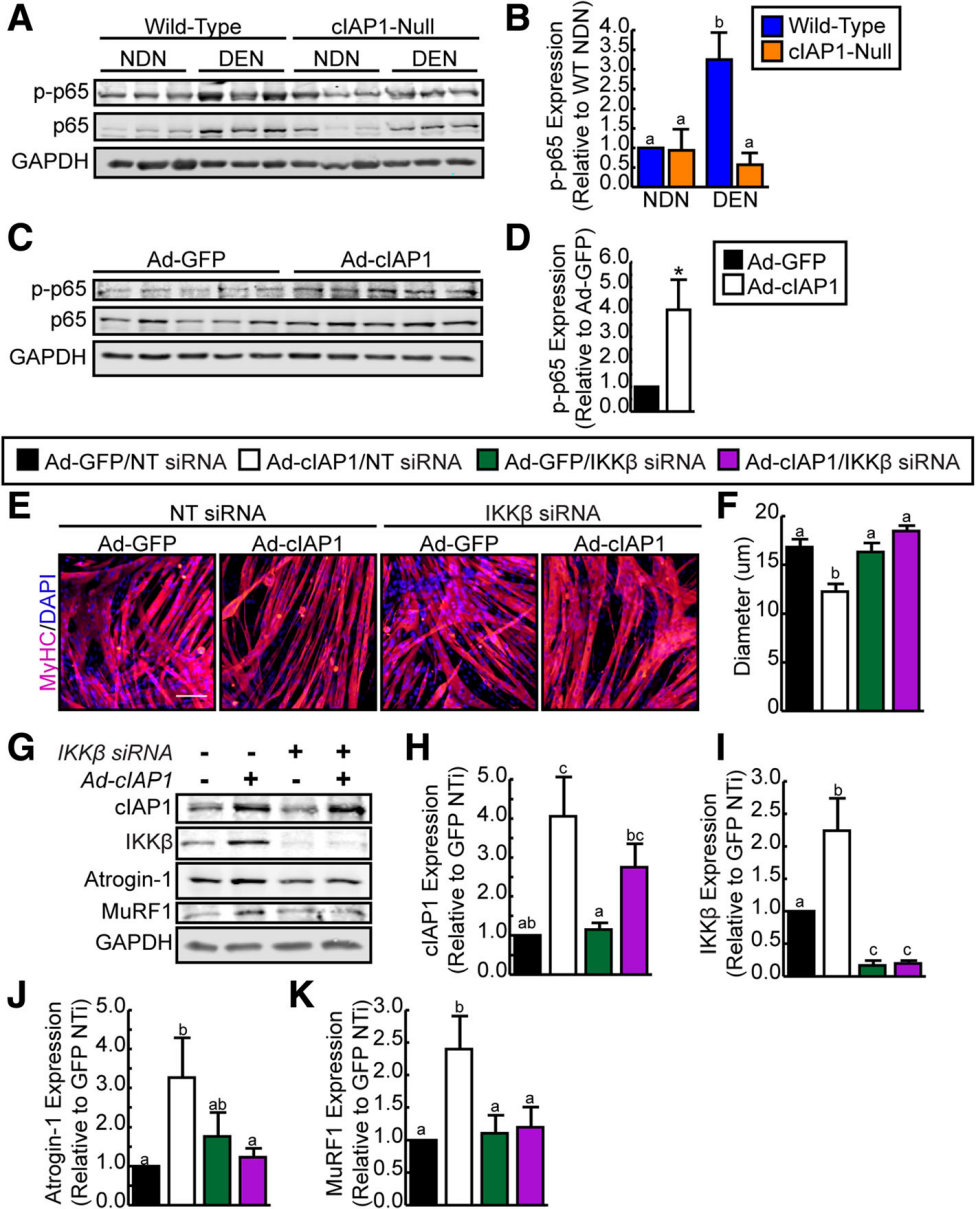
cIAP1 induces atrophy through upregulation of classical NF-κB signaling. **a** Representative Western blots of phospho-p65, p65, and GAPDH expression in gastrocnemius muscle from non-denervated (NDN) and denervated (DEN) hind limbs of C57BL/6 and cIAP1-null mice. **b** Quantification of phospho-p65 expression normalized with total protein and relative to wild-type non-denervated (WT NDN) muscle (*n* = 3). **c** Representative Western blots of phospho-p65, p65, and GAPDH expression in C2C12 myotubes infected with adenovirus expressing GFP (Ad-GFP) or human cIAP1 (Ad-cIAP1) for 24 h. **d** Quantification of phospho-p65 expression normalized with total protein and relative to Ad-GFP infected myotubes (*n* = 5). **e** Nascent (DM day 2) C2C12 myotubes were transfected with non-targeting (NT) siRNA or siRNA targeting murine IKKβ, allowed to form mature myotubes (DM day 4) and then infected with Ad-GFP or Ad-cIAP1 for 24 h. Myotubes were stained with MyHC (pink) and nuclei were counterstained with DAPI (blue). Representative images are shown. Scale bar = 50 um. **f** Myotube diameter of myotubes stained as in **e** (*n* = 5). **g** Representative Western blots of cIAP1, IKKβ, Atrogin-1, MuRF1, and GAPDH expression in cells cultured as described in **e**. Quantification of **h** cIAP1, **i** IKKβ, **j** Atrogin-1, and **k** MuRF1 protein expression normalized with total protein and relative to NT siRNA transfected/Ad-GFP infected C2C12 myotubes (*n* = 5). Data is the mean ± SEM, *p < 0.05, means with no common letters are significantly different from each other (*p* < 0.05)

NF-κB is a transcription factor that has been shown to be chronically activated in a number of muscle diseases including denervation-induced atrophy [5, 6]. Since cIAP1 is a critical positive regulator of NF-κB signaling in the skeletal muscle [22–24] and since cIAP1 is upregulated in the denervated muscle, we examined whether NF-κB signaling could be mediating cIAP1-induced atrophy. Non-denervated and denervated gastrocnemius muscles were isolated from wild-type and cIAP1-null mice, and protein extracts were extracted and analyzed by Western blot (Fig. 5a). Interestingly, the levels of phosphorylated NF-κB subunit p65 were significantly upregulated in wild-type denervated muscle (3.2-fold) compared to non-denervated muscle, and this phosphorylation was blunted in cIAP1-null animals (Fig. 5a, b). We also examined whether overexpression of cIAP1 in C2C12 myotubes could induce phosphorylation of p65 by Western blot analysis. Indeed, the levels of phosphorylated NF-κB subunit p65 were significantly upregulated in myotubes infected with cIAP1 (fourfold) compared to those infected with GFP (Fig. 5c, d). These results suggest that cIAP1 is required for the activation of classical NF-κB signaling upon muscle denervation and can induce phosphorylation of p65 in C2C12 myotubes.

To examine whether the classical NF-κB pathway mediates the pro-atrophic effect of cIAP1, we transfected nascent C2C12 myotubes (at day 2 of differentiation) with non-targeting siRNA or siRNA targeting the NF-κB activating kinase, IKKβ. Two days after transfection, mature myotubes were infected with adenovirus expressing GFP or cIAP1 for 24 h. Myotubes were then fixed and stained for MyHC to assess myotube diameter. Consistent with our previous findings, overexpression of cIAP1 was sufficient to induce myotube atrophy as demonstrated by the reduced myotube diameter in Ad-cIAP1/NT siRNA (12 μm) cultures compared to GFP/NT siRNA cultures (16 μm) (Fig. 5e, f). Interestingly, when IKKβ expression was knocked down, cIAP1 was unable to induce atrophy in C2C12 myotubes (Fig. 5e, f), suggesting that activation of classical NF-κB signaling is required for cIAP1 mediated muscle atrophy in vitro.

Activation of NF-κB signaling is known to activate proteolysis in the muscle through upregulation of MuRF1 expression and activity [5]; thus, we examined whether upregulation of MuRF1 by cIAP1 is through upregulation of NF-κB activity. Western blot analysis confirmed overexpression of cIAP1 and knockdown of IKKβ (Fig. 5g–i). Interestingly, we found that overexpression of cIAP1 in myotubes significantly upregulated IKKβ expression (twofold) (Fig. 5g, i). Consistent with our previous result (Fig. 2c, d), MuRF1 and Atrogin-1 expression were significantly upregulated when myotubes were infected with cIAP1; however, this upregulation was inhibited in the absence of IKKβ (Fig. 5g, j, k). These results suggest that cIAP1 upregulates MuRF1, and to a lesser extent Atrogin-1 expression through the activation of the classical NF-κB signaling pathway.

### Pharmacological ablation of cIAP1 with a Smac mimetic compound attenuates denervation-induced atrophy

Smac mimetic compounds (SMCs) are cIAP antagonists that function by inducing the auto-ubiquitination of cIAP1/2, resulting in their rapid proteasomal degradation [29]. Since we found that cIAP1 is an important regulator of muscle atrophy, we examined whether the SMC, LCL161 could attenuate denervation-induced muscle atrophy. LCL161 is an orally-available monomeric SMC that binds to cIAP1 with high affinity and has been proven safe in humans [30].

**Fig. 6:**
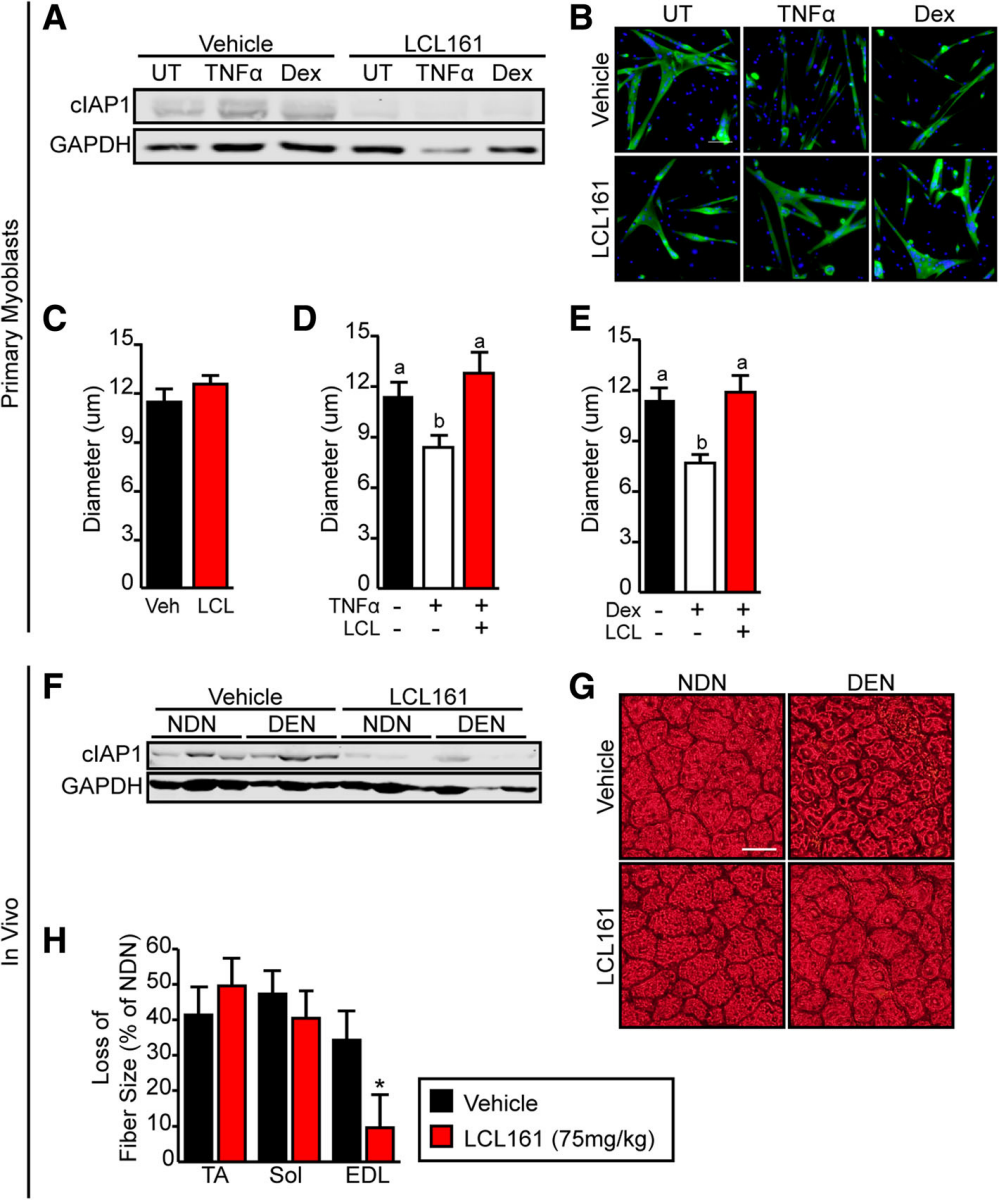
Pharmacological ablation of cIAP1 with LCL161 attenuates muscle atrophy. **a** Primary myoblasts were isolated from C57BL/6 mice and allowed to differentiate for 48 h to produce myotubes. Myotubes were treated with 10 ng/ml TNFα or 50 um dexamethasone (Dex) in the absence or presence of 500 nM LCL161. Untreated (UT) myotubes served as controls. Myotubes were collected for protein 24 h after treatment. Representative Western blots of cIAP1 and GAPDH expression. **b** Myotubes cultured as in **a** were stained with MyHC (green) and nuclei were counterstained with DAPI (blue). Representative images are shown. Scale bar = 50 um. Myotube diameter of MyHC+ myotubes in **c** UT vehicle and LCL161-treated cultures, **d** TNFα-treated cultures, and **e** Dex-treated cultures (*n* = 4). **f** C57BL/6 mice were treated with vehicle or LCL161 (75 mg/kg) at 4, 7, and 11 days after denervation of the right hind limb. Mice were sacrificed 14 days post-denervation. Representative Western blots of cIAP1 and GAPDH expression in the gas muscle. **g** Representative images of EDL muscle sections stained with H&E from mice described in **f**. Scale bar = 20 um. **h** Loss in TA, Sol, and EDL fiber size as a percentage of non-denervated control leg cross-sectional area (*n* = 4). Data is the mean ± SEM, *p < 0.05, means with no common letters are significantly different from each other (*p* < 0.05)

To examine if SMCs can lessen myotube atrophy in vitro, we isolated and differentiated myoblasts from wild-type mice and induced atrophy of myotubes (DM day 2) with TNFα or dexamethasone in the presence or absence of LCL161 for 24 h. We saw that both TNFα- and dexamethasone-treated myotubes had a higher cIAP1 expression in the absence of LCL161 (Fig. 6a). In the presence of LCL161, cIAP1 expression was eliminated in untreated and TNFα- and dexamethasone-treated myotubes (Fig. 6a). Inhibition of cIAP1 with LCL161 in myotubes did not promote myotube hypertrophy under basal conditions (Fig. 6b, c); however, LCL161 completely inhibited myotube atrophy in TNFα- and dexamethasone-treated myotubes (Fig. 6b, d, e) suggesting that inhibiting cIAP1 with SMCs can prevent myotube atrophy.

To examine whether LCL161 could inhibit muscle atrophy in vivo, the right hind limbs of wild-type mice were denervated and mice were treated with vehicle or LCL161 (75 mg/kg) at days 4, 7, and 11 post-denervation. Mice were examined 14 days post-denervation. LCL161 decreased cIAP1 protein expression in non-denervated muscle and denervated muscle (Fig. 6f). Furthermore, examination of the cross-sectional area showed that loss of fiber size (cross-sectional area) of the EDL was significantly reduced in LCL161-treated animals (9.6%) compared to vehicle-treated animals (34.2%) (Fig. 6g, h). Interestingly, pharmacological inhibition of cIAP1 did not appear to significantly attenuate denervation-induced atrophy in the TA and soleus muscles (Fig. 6h). These results taken together suggest that the use of SMCs to target cIAP1 for proteasomal degradation is a novel and promising therapeutic approach to treat muscle wasting diseases.

## Discussion

The present study provides novel insight into the mechanism of muscle atrophy and into the regulation of classical NF-κB signaling following denervation of the limb skeletal muscle. Our results demonstrate that the whole-body loss of cIAP1 (and not cIAP2), which is a critical, positive regulator of NF-κB signaling, can attenuate denervation-induced muscle atrophy as demonstrated by reduced muscle loss and decreased Atrogin-1 and MuRF1 expression. Histologically, the improvements were manifested particularly in the TA, gastrocnemius, and EDL. Interestingly, we did not see a significant attenuation of muscle wasting in the soleus of denervated hind limbs of cIAP1 mice. We have previously shown that loss of cIAP1 in *mdx* mice protected the soleus but not the EDL [23]. Contrary to that seen in Duchenne muscular dystrophy (DMD) in which fast-twitch fibers (EDL) are prone to damage, slow-twitch fibers (soleus) are more sensitive to sciatic nerve transection [31]. Further examination of the mechanisms underlying fiber type-specific atrophy would provide insight into novel strategies to prevent and treat muscle wasting diseases in which fast- and slow-twitch fibers are affected differently.

To investigate the molecular mechanism underlying the phenotype described above, we examined the activation of the UPS in denervated muscle of wild-type and cIAP1-null mice. Two muscle-specific E3 ubiquitin ligases, Atrogin-1 and MuRF1, have been identified that target muscle proteins for proteasomal degradation during muscle atrophy [3, 4]. Both Atrogin-1 and MuRF1 are upregulated in different muscle wasting conditions including denervation, and knockout mice for these genes are partially protected from the development of skeletal muscle atrophy [3, 4, 32]. Consistent with previous studies, we found that both MuRF1 and Atrogin-1 are upregulated in denervated muscle compared to non-denervated muscle. Moreover, we demonstrate that in the absence of cIAP1, MuRF1 consistently fails to be upregulated in denervated muscle, suggesting that one of the mechanisms by which loss of cIAP1 inhibits the degradation of muscle protein and confers protection against denervation-induced skeletal muscle atrophy is through blocking activation of the UPS.

Skeletal muscle wasting involves coordinated activation of a number of cell signaling pathways including NF-κB. Indeed, NF-κB signaling has been shown to be sufficient to induce skeletal muscle wasting in mice, in part by activating MuRF1 [5, 6]. Since we found that cIAP1 is sufficient to induce myotube atrophy in vitro and because cIAP1is an important regulator of NF-κB signaling, we examined whether cIAP1 activates the NF-κB/MuRF1 pathway during denervation-induced muscle atrophy. We found that loss of cIAP1 blunted activation of the NF-κB subunit p65 in denervated muscle and that overexpression of cIAP1 alone in wild-type myotubes increased activation of p65, suggesting that cIAP1 regulates classical NF-κB signaling in the skeletal muscle that leads to the atrophic state. Interestingly, when we inhibited NF-κB signaling with the use of a siRNA targeting IKKβ, cIAP1 was unable to induce myotube atrophy and MuRF1 expression, suggesting that during muscle atrophy, cIAP1 increases MuRF1 expression through activation of the classical NF-κB signaling pathway.

Further examination of interactions between cIAP1 and signaling factors in muscle wasting diseases would enhance our knowledge of the complex signaling networks regulating muscle atrophy. For example, cIAP1 may interact with TNF receptor-associated factor 6 (TRAF6), which was identified as an essential regulator of denervation-, cachexia-, and starvation-induced muscle atrophy [33–35]. In addition to activating NF-κB, TRAF6 was shown to mediate the activation of c-Jun N-terminal kinase 1/2 (JNK1/2), p38 mitogen-activated kinase (MAPK), and adenosine monophosphate-activated protein kinase (AMPK) in the skeletal muscle upon denervation [33]. Since cIAP1 is required to ubiquitinate TRAF6 and form a signaling scaffold [36, 37], it is possible that cIAP1 is essential for TRAF6-mediated effects on atrophy.

In summary, we observed that cIAP1 is upregulated in denervated skeletal muscle and that genetic loss of cIAP1 conferred resistance to denervation-induced muscle atrophy likely through inhibition of the IKKβ/NF-κB/MuRF1 pathway. Thus, this study identifies cIAP1 as a novel therapeutic target for denervation-induced muscle atrophy and may serve a similar role in other models of muscle atrophy including cancer cachexia, aging, and fasting. Indeed, we have shown that cIAP1 is upregulated in dystrophic muscle of mdx mice and that genetic loss of cIAP1 improved muscle pathology [23]. In this regard, potent cIAP1/2 small molecule inhibitors known as Smac-mimetic compounds (SMCs) could be used to pharmacologically inhibit cIAP1 in muscle diseases. SMCs function by inducing the auto-ubiquitination of cIAP1/2, resulting in their rapid proteasomal degradation [29], and have been shown to be well-tolerated in humans [30]. In this study, we show that the SMC, LCL161 can prevent upregulation of cIAP1 and attenuate muscle atrophy in vitro and in vivo. Here, we show that LCL161 can reduce the loss of fiber size in the EDL; however, further studies need to be completed to determine the best dosing strategy of SMCs for the use of muscle wasting diseases. Thus, the use of SMC treatment to prevent or delay muscle atrophy may constitute a relatively direct path from bench to bedside for the treatment of muscle wasting diseases.

## Conclusions

Although several studies have shown the major role of constitutive activation NF-κB signaling in muscle diseases [5, 6, 9, 33, 38], our study establishes cIAP1 as an important upstream regulator of this pathway in a model of denervation-induced muscle atrophy. The characterization of novel factors controlling muscle atrophy provide important insights into the molecular mechanisms regulating skeletal muscle disease and can fuel the development of novel therapeutic approaches for the treatment of muscle atrophies.

## Additional file

Additional file 1 : Figure S1 Genetic ablation of cIAP1 attenuates denervation-induced atrophy. A small (5–10 mm) piece of the right sciatic nerve was removed to induce denervation of the entire right hind limb of 6-week-old C57BL/6 mice (blue bars) or sex- and age-matched cIAP1-null mice (orange bars). The contralateral leg served as an internal control. Mice were sacrificed 7 days post-denervation. (A) Representative images of TA muscle sections stained with H&E from mice described above. (B) Decrease in TA fiber size as a percentage of non-denervated control leg cross-sectional area (*n* ≥ 2). Data is the mean ± SEM, n.s. not significant. (ZIP 350 kb)

## Data Availability

Data sharing is not applicable to this article as no datasets were generated or analyzed during the current study.

## Abbreviations

ALS: Amyotrophic lateral sclerosis
BIR: Baculovirus IAP repeat
cIAP1/2: Cellular IAP1/2
DEN: Denervated
EDL: Extensor digitorum longus
Gas: Gastrocnemius
GFP: Green fluorescent protein
H&E: Hematoxylin and eosin
IAP: Inhibitors of apoptosis
IKKβ: Inhibitor of NF-κB kinase subunit beta
MAFbx1: Muscle atrophy F-Box 1
MuRF1: Muscle RING finger-1
MyHC: Myosin heavy chain
NDN: Non-denervated
NF-κB: Nuclear factor kappa-light-chain-enhancer of activated B cells
RING: Really interesting new gene
SMA: Spinal muscle atrophy
SMCs: Smac-mimetic compounds
Sol: Soleus
TA: Tibialis anterior
TNFα: Tumor necrosis factor alpha
UPS: Ubiquitin-proteasome system
