# Supplementary figures and images for: Targeted ablation of the cellular inhibitor of apoptosis 1 (cIAP1) attenuates denervation-induced skeletal muscle atrophy
Source: Skelet Muscle. 2019 May 24;9:13. doi: 10.1186/s13395-019-0201-6 (PMC6533726; doi:10.1186/s13395-019-0201-6)

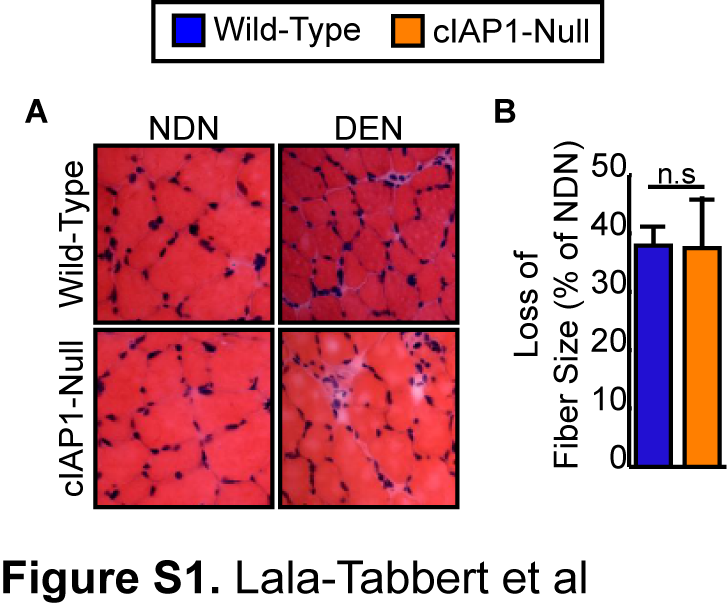

Supplement: Supplementary file 1 — : Figure S1 Genetic ablation of cIAP1 attenuates denervation-induced atrophy. A small (5–10 mm) piece of the right sciatic nerve was removed to induce denervation of the entire right hind limb of 6-week-old C57BL/6 mice (blue bars) or sex- and age-matched cIAP1-null mice (orange bars). The contralateral leg served as an internal control. Mice were sacrificed 7 days post-denervation. (A) Representative images of TA muscle sections stained with H&E from mice described above. (B) Decrease in TA fiber size as a percentage of non-denervated control leg cross-sectional area (n ≥ 2). Data is the mean ± SEM, n.s. not significant. (ZIP 350 kb) [file 13395_2019_201_MOESM1_ESM.zip › Figure S1..tif]
